# Research on hand, foot and mouth disease incidence forecasting using hybrid model in mainland China

**DOI:** 10.1186/s12889-023-15543-9

**Published:** 2023-03-31

**Authors:** Daren Zhao, Huiwu Zhang, Ruihua Zhang, Sizhang He

**Affiliations:** 1 Department of Medical Administration, Sichuan Provincial Orthopedics Hospital, Chengdu, Sichuan People’s Republic of China; 2 School of Management, Chengdu University of Traditional Chinese Medicine, Chengdu, Sichuan People’s Republic of China; 3 General Practitioners Training Center of Sichuan Province, Chengdu, Sichuan People’s Republic of China; 4 Department of Information and Statistics, The Affiliated Hospital of Southwest Medical University, Luzhou, 64600 Sichuan China

**Keywords:** Prediction model, SARIMA model, SARIMA-BPNN hybrid model, SARIMA-PSO-BPNN hybrid model

## Abstract

**Background:**

This study aimed to construct a more accurate model to forecast the incidence of hand, foot, and mouth disease (HFMD) in mainland China from January 2008 to December 2019 and to provide a reference for the surveillance and early warning of HFMD.

**Methods:**

We collected data on the incidence of HFMD in mainland China between January 2008 and December 2019. The SARIMA, SARIMA-BPNN, and SARIMA-PSO-BPNN hybrid models were used to predict the incidence of HFMD. The prediction performance was compared using the mean absolute error(MAE), mean squared error(MSE), root mean square error (RMSE), mean absolute percentage error (MAPE), and correlation analysis.

**Results:**

The incidence of HFMD in mainland China from January 2008 to December 2019 showed fluctuating downward trends with clear seasonality and periodicity. The optimal SARIMA model was SARIMA(1,0,1)(2,1,2)_[12]_, with Akaike information criterion (AIC) and Bayesian Schwarz information criterion (BIC) values of this model were 638.72, 661.02, respectively. The optimal SARIMA-BPNN hybrid model was a 3-layer BPNN neural network with nodes of 1, 10, and 1 in the input, hidden, and output layers, and the R-squared, MAE, and RMSE values were 0.78, 3.30, and 4.15, respectively.

For the optimal SARIMA-PSO-BPNN hybrid model, the number of particles is 10, the acceleration coefficients c1 and c2 are both 1, the inertia weight is 1, the probability of change is 0.95, and the values of R-squared, MAE, and RMSE are 0.86, 2.89, and 3.57, respectively.

**Conclusions:**

Compared with the SARIMA and SARIMA-BPNN hybrid models, the SARIMA-PSO-BPNN model can effectively forecast the change in observed HFMD incidence, which can serve as a reference for the prevention and control of HFMD.

**Supplementary Information:**

The online version contains supplementary material available at 10.1186/s12889-023-15543-9.

## Background

Hand, foot, and mouth disease (HFMD) is an acute infectious disease caused by EV71 and the Cox A16 enterovirus, which spreads globally and is prevalent among children under five years of age [1–3]. HFMD is transmitted primarily through contact with the gastrointestinal and respiratory tracts and close contacts, and can develop throughout the year [4]. Most children with HFMD have mild symptoms, but a small percentage of infected individuals can develop severe disease [5, 6]. HFMD is a self-limiting disease that mainly manifests as fever and herpes on the hands, feet, and mouth [7]. Few children develop complications, such as myocarditis, pulmonary edema, and meningoencephalitis [7, 8]. HFMD is also a global infectious disease, and its prevalence has been reported in most regions of the world, especially in the Asia–Pacific and Western Pacific Region [3, 9]. It has been reported that 96,900 Disability Adjusted life years per year are due to HFMD in some countries in East and Southeast Asia, and HFMD causes a more severe economic burden of disease in these countries [10].

HFMD is not only a public health issue of global concern but has also become a widespread and typical infectious disease in mainland China. Several large outbreaks of HFMD occurred in 2007 and early 2008 in China; therefore, HFMD was included in the reporting of category C infectious diseases on May 2, 2008 [11]. Since HFMD was included in the management of category C infectious diseases of the Chinese Communicable Diseases Control Law, the number of cases of HFMD incidence and deaths has been ranked at the top of the list of legally reported infectious diseases in mainland China [12]. An average of approximately 2 million cases have been reported each year in 31 provinces and municipalities of mainland China [13]. HFMD causes a greater economic burden of disease in mainland China. A study on the economic burden of HFMD in mainland China showed that the average per capita cost of HFMD cases during treatment was 600–1,000 RMB for mild outpatient cases, 3,000–5,000 RMB for general inpatient cases, and 15,000–25,000 RMB for severe cases (without considering their impact on social productivity) [14]. Moreover, it is estimated that the direct economic burden of all severe HFMD cases in Jiangsu Province, China, was RMB 16.64 million during 2017–2018 [15]. Therefore, prevention and control of HFMD continues to be an important public health issue in mainland China.

Early surveillance and warning of HFMD are of high priority and important work. If the government and related departments can effectively monitor and provide accurate early warnings of HFMD, they will be able to respond in advance and provide information for the proper allocation of medical resources [16]. Therefore, strengthening the surveillance and prediction of HFMD epidemiological trends in China is important for implementing effective preventive and control measures. Exploring approaches to enhance early monitoring and warning capabilities has become an urgent priority for improving China’s public health system.

Many scholars have conducted extensive research on predicting the incidence of HFMD. Because the incidence of HFMD presents obvious seasonal and periodic characteristics, most current studies have focused on using the traditional time series auto-regressive integrated moving average (ARIMA) model for forecasting. Although the ARIMA model has achieved a better performance in predicting the incidence of HFMD [17, 18], there is still a failure to fully mine the nonlinear information of seasonal infectious disease data [19]. Some studies have focused on using machine learning models to predict the incidence of HFMD [20–22], but they may not explain the nonlinear functions within the time-series data in practice [23]. Moreover, a few studies have combined traditional time-series ARIMA models with machine learning models to develop hybrid models that have achieved better prediction performance [16, 23, 24]. However, hybrid models only combined the advantages of the two models, and there may be insufficient optimization of the model parameters. Therefore, the prediction performance of these models needs to be further improved.

To overcome the shortcomings of a single SARIMA for nonlinear information processing and the hybrid model with insufficiently optimized parameters, in the present study, we first proposed a SARIMA-PSO-BPNN hybrid model for forecasting the incidence of HFMD between January 2008 and December 2019 in mainland China. We constructed the SARIMA and SARIMA-BPNN hybrid models based on the data characteristics of HFMD incidence in mainland China and optimized the SARIMA-BPNN hybrid model using the Particle Swarm Optimization (PSO) algorithm. Predictions from the SARIMA-PSO-BPNN hybrid model can serve as an information reference for the surveillance and early warning of HFMD in mainland China.

## Methods

### Data source

Data on monthly HFMD incidence from January 2008 to December 2018 in 31 provinces and municipalities in mainland China were obtained from the China Public Health Science Data Center website (https://www.phsciencedata.cn/Share/index.jsp). The total number of HFMD cases from January to December 2019 was obtained from the National Health Commission of the People’s Republic of China’s website (http://www.nhc.gov.cn/jkj/pgzdt/new_list.shtml). The overall population size in 2019 was obtained from the Chinese Statistical Yearbook (http://www.stats.gov.cn/tjsj/ndsj/2021/indexch.htm). The average population per year was calculated as the population at the beginning and end of the year.

A total of 144 data on the monthly incidence of HFMD in mainland China from 2008 to 2019 were included in this study. We divided the HFMD incidence data into training and test sets. HFMD incidence data from January 2008 to December 2018 were used as the training set to construct the models, and data from January to December 2019 were used as the test set to evaluate the generalization capability of the models.

### SARIMA model

Auto-regressive integrated moving average (ARIMA) model is a well-known time-series forecasting method proposed by Box and Jenkins in the early 1970s, also known as the Box-Jenkins model [25]. If the time series contains significant seasonal characteristics, the model can be identified as a SARIMA model. The SARIMA model is expressed as SARIMA (p, d, q) (P, D, Q)s and can be expressed as [26, 27]:

1 $$\nabla^{d} \nabla_{S}^{D} Y_{t} = \frac{{\theta_{q} (B)\Theta Q(B^{S} )}}{{\phi_{p} (B)\Phi P(B^{S} )}}\varepsilon_{t}$$

2 $$\phi_{p} (B) = 1 - \phi_{1} {\text{B}} - \phi_{2} {\text{B}}^{{2}} - \phi_{3} {\text{B}}^{{3}} - ...\phi_{p} {\text{B}}^{{\text{p}}}$$

3 $$\theta_{q} (B) = 1 - \theta_{1} {\text{B}} - \theta_{2} {\text{B}}^{{2}} - \theta_{3} {\text{B}}^{{3}} - ...\theta_{q} {\text{B}}^{{\text{q}}}$$

4 $$\Phi P(B^{S} ) = 1 - \Phi_{1} B^{S} - \Phi_{2} B^{2s} - \Phi_{3} B^{3s} - ...\Phi_{P} B^{Ps}$$

5 $$\Theta {\text{Q}}(B^{S} ) = 1 - \Theta_{1} B^{S} - \Theta_{2} B^{2s} - \Theta_{3} B^{3s} - ...\Theta_{P} B^{{{\text{Q}}s}}$$

where p, q, P, and Q denote the order of auto-regression, the order of moving average, seasonal auto regression lag, and seasonal moving average, respectively. D and d denote the degree of seasonal and degree of trend differences, respectively, and s denotes the length of the seasonal period. Where B is the backward shift operator, Y_t_ is the HFMD incidence time-series at time t, and ε_t_ is the residual of the HFMD incidence time-series. Where $\phi_{p}$ is the p-order auto-regressive coefficient polynomial, $\theta_{q}$ is the q-order moving average coefficient polynomial, $\Phi P(B^{S} )$ is the seasonal polynomial function of order P, and $\Theta {\text{Q}}(B^{S} )$ is the seasonal polynomial function of order Q.

Four major steps are involved in the construction of the SARIMA model [19, 28]. The first step was to determine whether the time series were stationary. In general, the stationary of a time series is determined by plotting the original time series chart or using methods such as Augmented Dickey-Fuller (ADF) tests. If the time series is non-stationary, it must be converted into a stationary time series using a difference or logarithmic transformation. The second step is to identify the parameters of the SARIMA model. The possible parameters of p, q, P, and Q were initially determined by plotting auto-correlation function (ACF) and partial auto-correlation function (PACF) charts. We then initially fitted the candidate SARIMA models based on the possible parameters p, q, P,and Q. The third step was to conduct model diagnosis. Residual tests were performed using the Ljung-Box Q test. Statistical significance of the model parameters was assessed using *t*-test and *p*-value. The fourth step is to identify the parameters of the SARIMA model and select the optimal model. The optimal model was selected based on the white noise residuals and the lowest AIC and BIC values.

### BPNN model

A backward propagation neural network (BPNN) is a multilayer feedforward neural network with output results using forward propagation and errors using backward propagation [29]. The main working principle of the BPNN is to use machine learning to continuously iterate the training model, calculate the error between the actual and expected output values based on the minimum mean squared error criterion, and adjust the weights and thresholds of each layer of the network using the gradient descent strategy to minimize the error [30]. A classical BPNN is a 3-layer neural network consisting of an input layer, hidden layer, and output layer with fully interconnected neurons between adjacent layers and unconnected neurons within the same layer [21].

**Fig. 1:**
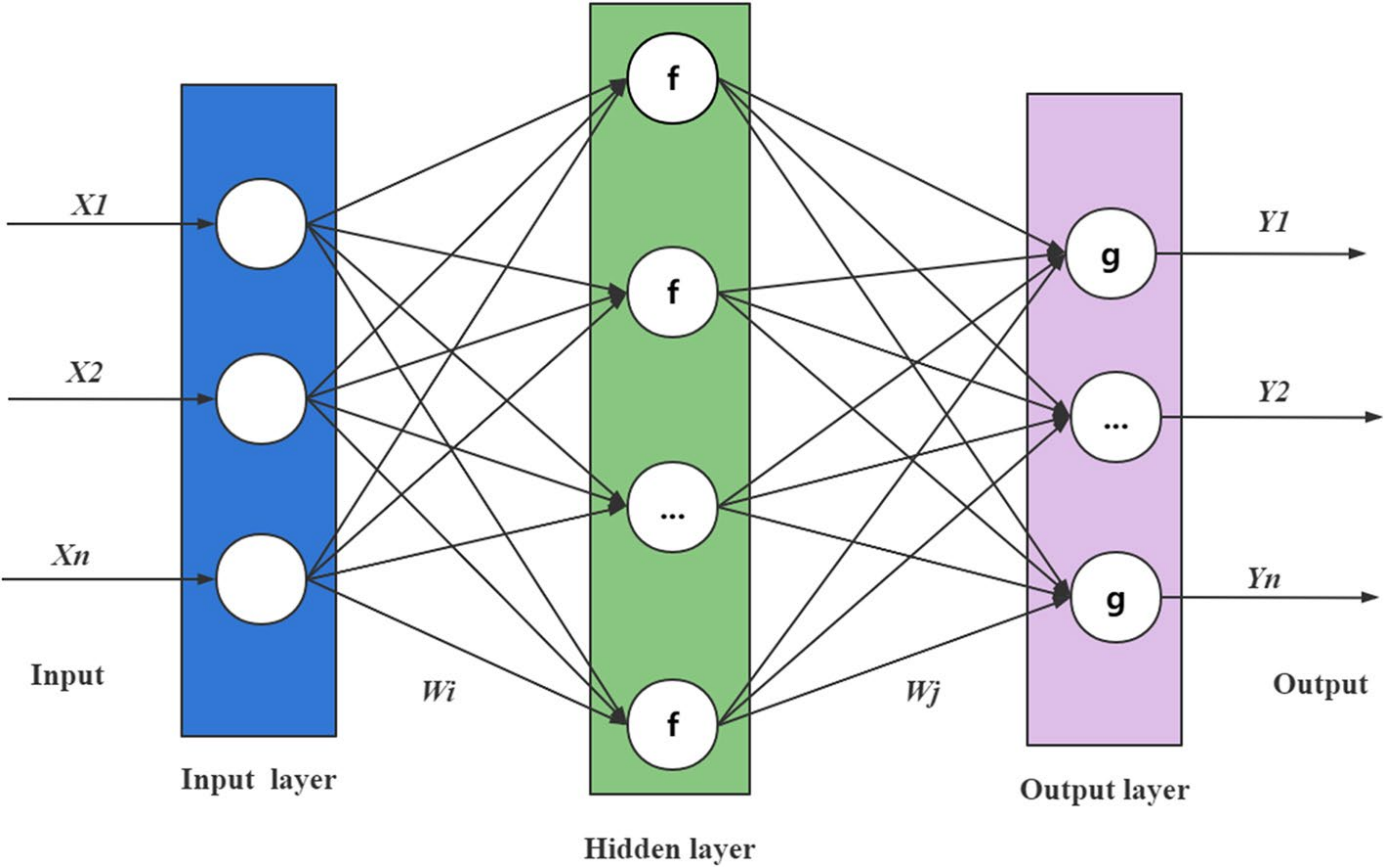
The basic structure of a BP neural network

There are three main steps in BPNN modeling [21, 31]: (1) initialization of the network and setting of network parameters, (2) normalization of the original data, dividing the training and test sets of the data, and back-propagation of the associated error calculation and adjustment of thresholds and weights, and (3) inverse normalization of the data to obtain the predicted values. The basic structure of a BPNN is shown in Fig. 1.

As shown in Fig. 1, we set up a 3-layer neural network with an input layer, hidden layer, and output layer. Assume that the input vector A = [X_1_, X_2_, X_3_,..., Xi], hidden layer input vector F = [f_1_, f_2_, f_3_,..., f_n_], output layer input vector G = [g_1_, g_2_, g_3_,..., gj], and actual output vector Y = [ Y_1_, Y_2_,Y_3_,..., Y_k_].

The hidden layer output (H_i_) is expressed as [32, 33]:

6 $${\text{H}}_{{\text{i}}} = f\left( {\sum\limits_{i = 1}^{n} {{\text{W}}_{ij} - \theta_{j} } } \right)\begin{array}{*{20}c} {} & {j = 1,2,...,n} \\ \end{array}$$

where i is the number of hidden nodes, W_ij_ is the connection weight of the input layer unit i to hidden layer unit j, θ_j_ is the threshold from the input layer to the hidden layer, and f is the excitation function.

7 $$f(x) = \frac{1}{1 + \exp ( - x)}$$

The prediction output (O_k_) is expressed as:

8 $${\text{O}}_{{\text{k}}} = \sum\limits_{j = 1}^{l} {H_{{\text{i}}} } {\text{W}}_{jk} - \theta_{k}$$

where W_jk_ is the connection weight of the hidden layer unit j to the output layer unit k and θ_k_ is the threshold from the hidden layer to the output layer.

And then the prediction error e is expressed as:

9 $${\text{e}}_{{\text{k}}} = Y_{k} - O_{k}$$

where O_k_ is the prediction output, and Y_k_ is the actual output.

The number of hidden layer nodes was calculated using Eq. (10).

10 $$h = \sqrt {m + n} + a$$

where h is the number of hidden layer nodes and m and n are the numbers of input layer nodes and output layer nodes, respectively. Where a is the adjustment constant between 1 and 10.

### SARIMA-BPNN hybrid model

The SARIMA-BPNN hybrid model was developed similarly to that of the BPNN model. The modeling steps of the SARIMA-BPNN hybrid model are as follows: (1) The optimal SARIMA model was constructed using the HFMD incidence data from January 2008 to December 2018 in mainland China. (2) A 3-layer BPNN model was constructed using the predicted values from the optimal SARIMA model as the input variables and the observed value of the HFMD incidence data as the output variable. (3) According to the modeling steps of the BPNN model, the data were divided into a training set (70% of the data) and a test set (30% of the data) and then normalized before constructing the model. (4) The model with the trained BPNN was then simulated and the data obtained from the simulation were back-normalized to obtain the predicted values. The optimal SARIMA-BPNN hybrid model was determined by the largest R-squared value and lowest MAE and RMSE values.

### SARIMA-PSO-BPNN hybrid model

Particle Swarm Optimization (PSO) is a heuristic search technique with simple implementation, high global search capability, and superior performance [34]. PSO simulates the foraging behavior of birds and is used to solve optimization problems [35]. PSO was introduced into the BPNN model to accelerate the convergence of the traditional BPNN algorithm. The main modeling steps of the SARIMA-PSO-BPNN hybrid model are as follows [36]: (1) The parameters of the PSO algorithm were set based on the established SARIMA-BPNN hybrid model. The size of the population, variable range, inertia weight, learning factor, and maximum number of iterations were determined by many attempts. (2) Based on Eq. (11), the fitness value of each particle is calculated as follows [36]:

11 $$fitness = \frac{1}{1 + E}$$

where E is the training error precision.

And then, according to Eq. 12, the velocity and position of the particle are updated.

12 $$v_{i + 1} (t + 1) = \omega v_{i} (t) + {\text{c}}_{1} r_{1} (pbest_{i} (t) - x_{i} (t)) + {\text{c}}_{2} r_{2} (gbest_{i} (t) - x_{i} (t)),x_{i + 1} (t + 1) = x_{i} (t) + v_{i + 1} (t + 1)$$

where pbest and gbest are the best particle and swarm positions, respectively. x_i_ is the position vector; v_i_ is the velocity vector; c_1_ and c_2_ are the learning factors; and r_1_ and r_2_ are random values between 0 and 1. (3) The PSO-optimized weights and thresholds were substituted into the BPNN. The neural network optimized using PSO was trained using training samples until the error requirement was satisfied. Finally, an optimal SARIMA-PSO-BP hybrid model was constructed.

### Evaluation of prediction performance

The mean absolute error (MAE), mean squared error (MSE), root mean square error (RMSE), mean absolute percentage error (MAPE), and correlation analyses were applied to comprehensively evaluate the prediction performance of the SARIMA, SARIMA-BPNN, and SARIMA-PSO-BPNN hybrid models. The smaller the values of MAE, MSE, RMSE, and MAPE, the better is the prediction performance of the model [37]. These indicators are expressed as follows:

13 $${\text{MAE}} = \frac{{\sum\limits_{t = 1}^{n} {\left| {X_{t} - {\hat{\text{X}}}_{t} } \right|} }}{n}$$

14 $${\text{MSE}} = \frac{1}{n}\sum\limits_{t = 1}^{n} {(X_{t} - \hat{X}_{t} )^{2} }$$

15 $${\text{RMSE}} = \sqrt {\frac{{\sum\limits_{t = 1}^{n} {(X_{t} - {\hat{\text{X}}}_{t} )^{2} } }}{n}}$$

16 $${\text{MAPE}} = \frac{1}{n}\sum\limits_{t = 1}^{n} {\left| {\frac{{X_{t} - {\hat{\text{X}}}_{t} }}{{X_{t} }}} \right|}$$

where ${\hat{\text{X}}}_{t}$ is the predicted value, $X_{t}$ is the observed value of the monthly HFMD incidence, and n is the sequence sample size.

Pearson’s and Spearman’s correlation coefficients were used to test the correlation between the predicted values of each model and observed values. A correlation coefficient with an absolute value closer to 1 indicates a stronger correlation between two variables [38, 39]. The strength of the correlation was evaluated [38, 39] as shown in Table 1.

**Table 1:** Correlation strength judgment

Pearson		Spearman	
Coefficients	Interpretation	Coefficients	Interpretation
0.00–0.10	Negligible correlation	0.00–0.30	Negligible correlation
0.10–0.39	Weak correlation	0.30–0.50	Weak correlation
0.40–0.69	Moderate correlation	0.50–0.70	Moderate correlation
0.70–0.89	Strong Correlation	0.70–0.90	Strong Correlation
0.90–1.00	Very Strong Correlation	0.90–1.00	Very Strong Correlation

### Data analysis

R software (version 4.1.1) was used to construct the SARIMA model, and MATLAB software (Version R2020b, MathWorks, Natick, MA, USA) was used to construct the SARIMA-BPNN and SARIMA-PSO-BPNN hybrid models. The level of significance was set at *p* < 0.05.

## Results

### General description

**Fig. 2:**
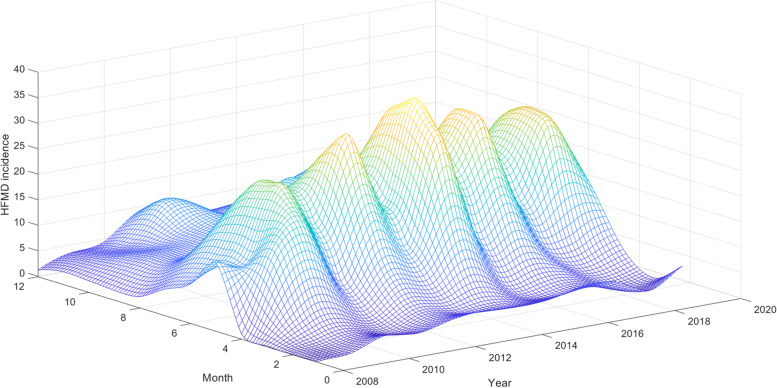
Three-dimensional time series of HFMD incidence in China from January 2008 to December 2021

As shown in Fig. 2, the incidence of HFMD time series presented clear seasonality and periodicity patterns. The HFMD incidence rate increased at an average monthly rate of 2.56% from January 2008 to December 2019, with a peak incidence between May and June each year and a low incidence between January and February each year.

### SARIMA model

**Fig. 3:**
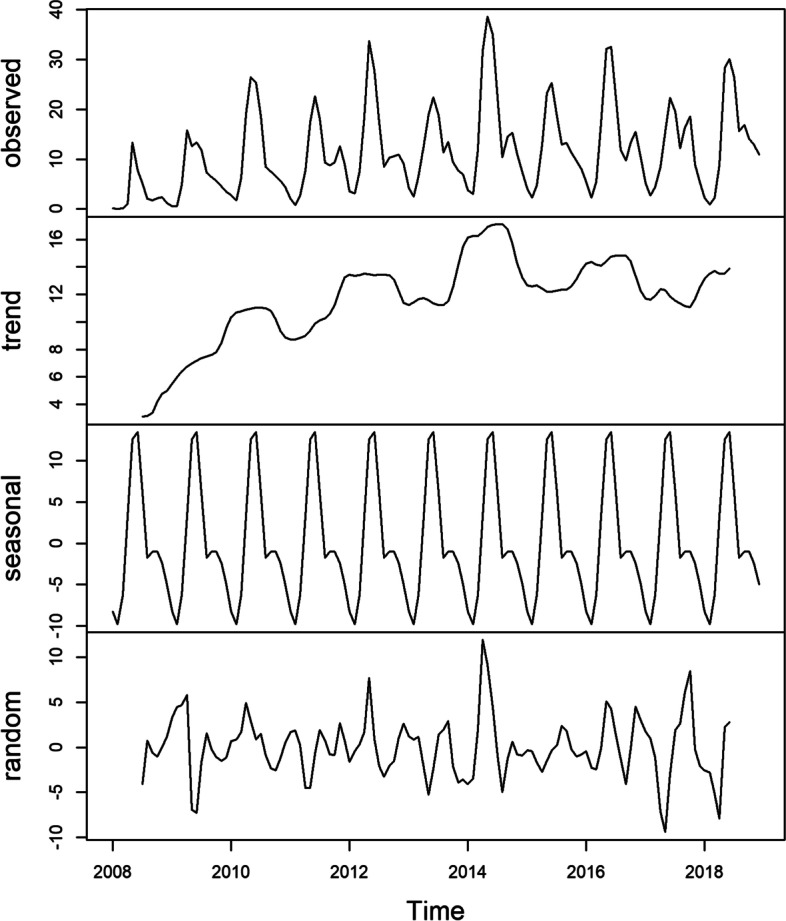
Decomposition of additive HFMD time series from January 2008 to December 2021 in mainland China

The decomposition () function in the R software was used to decompose the time series of HFMD incidence data from January 2008 to December 2019 in mainland China into seasonal, trend, and random components (Fig. 3). The results reconfirmed that the time series of HFMD incidence data from January 2008 to December 2019 in China had obvious seasonality, trends, periodicity, and randomness.

**Fig. 4:**
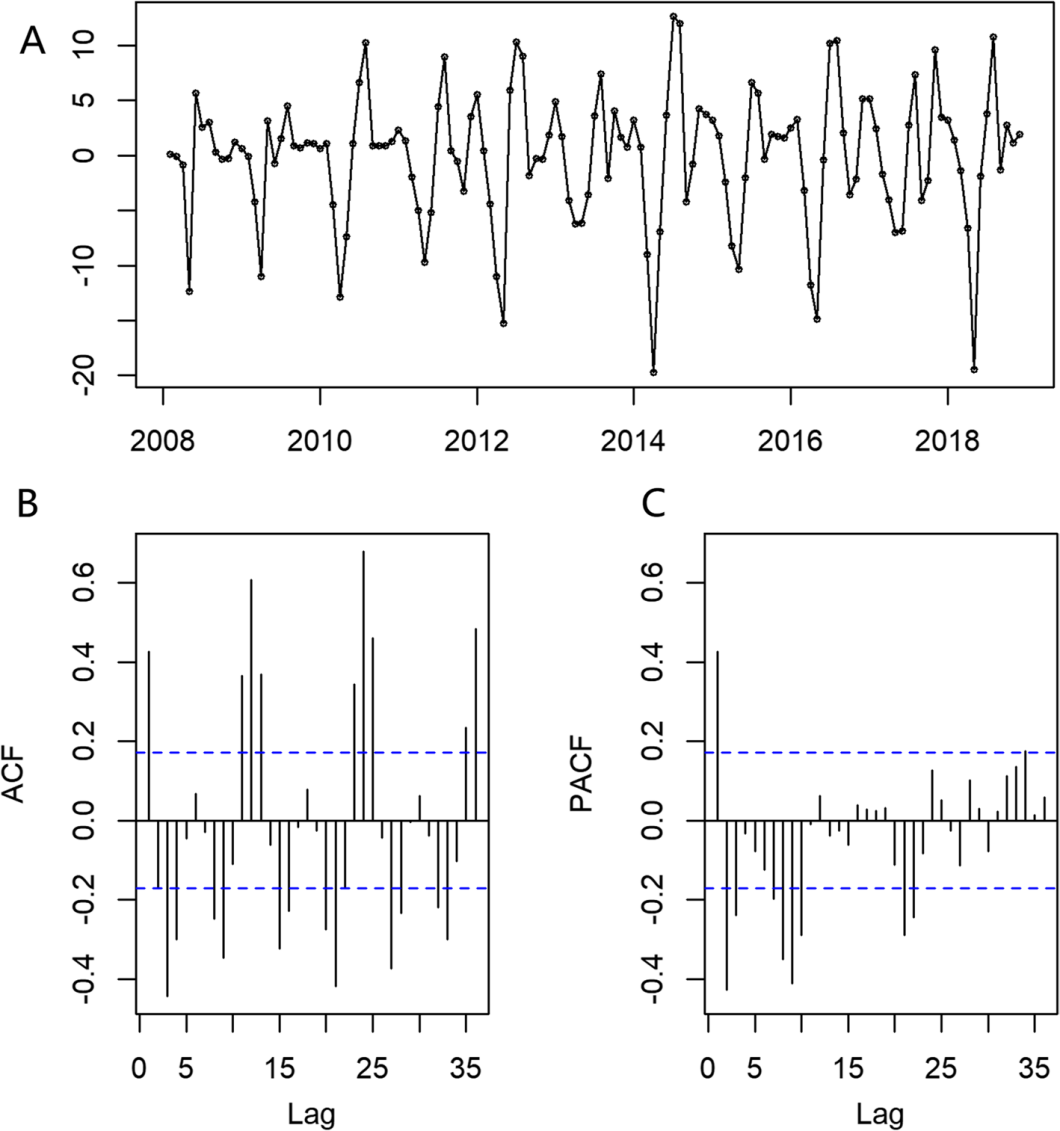
Differenced HFMD time series, ACF, and PACF plots (**A**) Differenced HFMD time series. **B** ACF plot of Differenced HFMD time series. **C** PACF plot of Differenced HFMD time series

After a difference in the original HFMD data, the transformation time series became stationary. The Augmented Dickey-Fuller (ADF) test also confirmed this result (*t* = -6.264, *p* = 0.01); thus, d was 0 or1 and D was1 (Fig. 4A). The ACF diagram shows an obvious peak at lag 1; thus, the value of the non-seasonal p may be 1. In addition, a spike at 12 in the first cycle indicated that the value of seasonal Q may be 1 or 2 (Fig. 4B). Similarly, the PACF plot showed a trailing trend at lag 2; thus, the value of seasonal P may be 2, and a spike at 1or 2 in the first cycle indicates that non-seasonal q may be 1or 2 (Fig. 4C). After the initial determination of the model parameters using ACF and PACF diagrams, the candidate SARIMA models were constructed (Table 2). The optimal SARIMA model was SARIMA(1,0,1)(2,1,2)_[12]_, which had the lowest AIC and BIC values. All four candidate SARIMA models passed the Ljung-Box Q test, indicating that the residual series of these models was white-noise (Table 2). We then used the SARIMA(1,0,1)(2,1,2)_[12]_ model to predict the HFMD incidence data from January to December 2019 in mainland China.

**Table 2:** The candidate SARIMA models and Ljung-Box Q test

Candidate Models	AIC	BIC	Ljung-Box Q Statistics	*p-value*
SARIMA(1,1,1)(2,1,1)_[12]_	654.46	671.13	0.056	0.811
SARIMA(1,1,1)(2,1,2)_[12]_	652.94	672.39	0.060	0.805
SARIMA(1,0,1)(2,1,2)_[12]_	638.72	661.02	0.0004	0.984
SARIMA(1,0,2)(2,1,1)_[12]_	645.82	665.33	0.187	0.665
SARIMA(1,0,2)(2,1,2)_[12]_	644.78	667.07	0.170	0.680

### SARIMA-BPNN hybrid model

The number of neural nodes in the BPNN hidden layer was calculated using Eq. (10) to yield a range of values of h between 2 and 11. Through repeated experiments, we set the number of nodes in the input, hidden, and output layers to 1, 10, and 1, respectively, and the epochs, learning rate, and minimum error of the training target to 1000, 0.01, and 0.00001, respectively, to obtain an optimal BPNN model. The R-squared, MAE, and RMSE values of the model were 0.78, 3.30, and 4.15, respectively.

### SARIMA-PSO-BPNN hybrid model

**Fig. 5:**
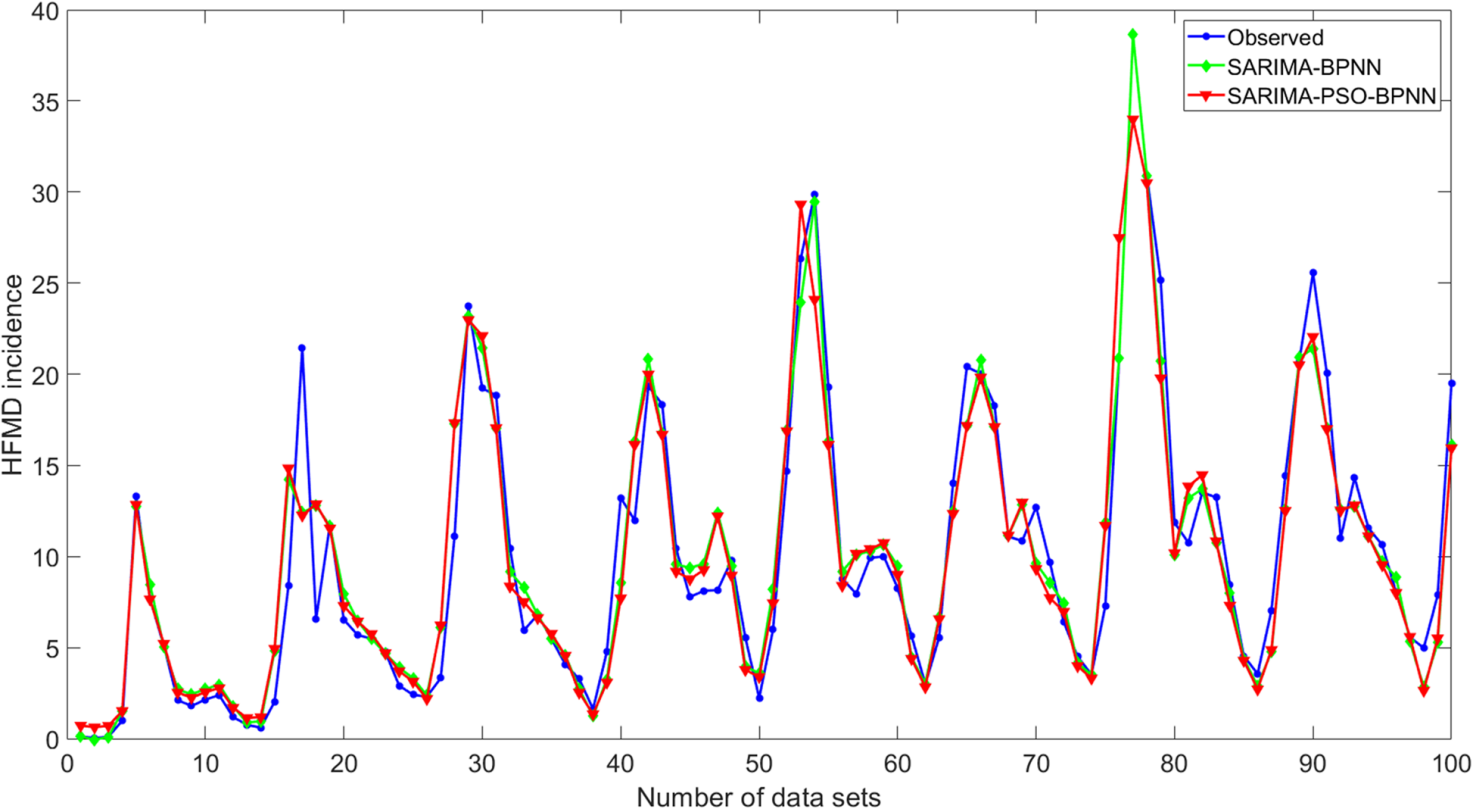
Comparison of observed and predicted values from SARIMA-BPNN and SARIMA-PSO-BPNN hybrid models in the training set

**Fig. 6:**
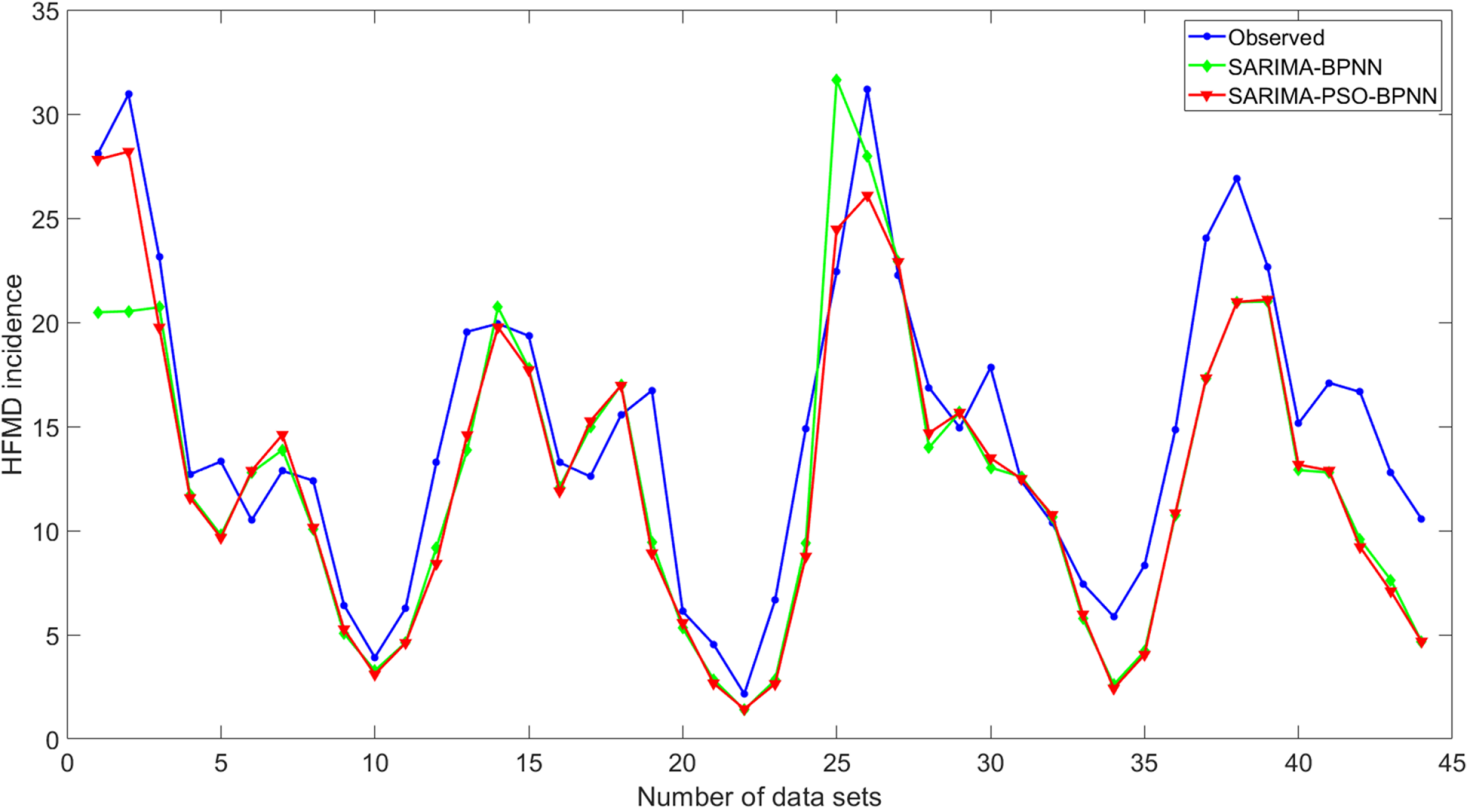
Comparison of observed and predicted values from SARIMA-BPNN and SARIMA-PSO-BPNN hybrid models in the test set

Based on the SARIMA-BPNN hybrid model, the SARIMA-PSO-BPNN hybrid model was constructed using the PSO algorithm. Therefore, the network structure of the SARIMA-PSO-BPNN hybrid model was consistent with that of the SARIMA-BPNN hybrid model. The number of population updates was set to 50, the population size to 10, the acceleration coefficient c1 = c2 = 1, the maximum velocity Vmax to 2, the minimum velocity Vmin to -1, popmax = [100, 10], popmin = [0.1, 0.6], inertia weight to 1, and a probability of variation of 0.95, the optimal SARIMA-PSO-BPNN hybrid model was obtained. The R-squared, MAE, and RMSE values of the model were 0.86, 2.89, and 3.57, respectively. The predictions from the SARIMA-BPNN and SARIMA-PSO-BPNN hybrid models for the training and test sets are shown in Figs. 5 and 6.

### Prediction performance

**Fig. 7:**
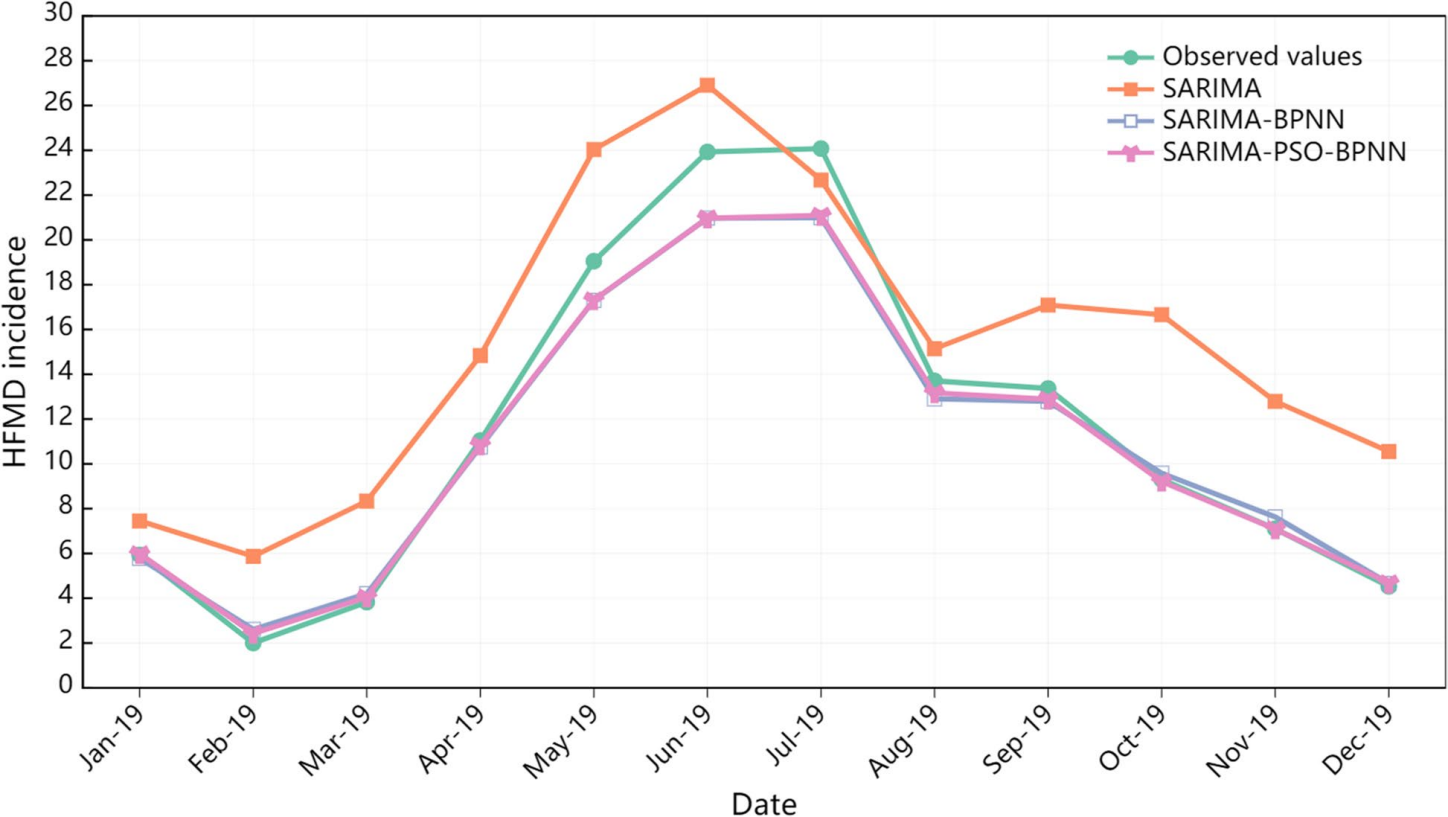
Predictive performance of the three models

In the training set, except for the MAPE value, the MAE, MSE, and RMSE values of the SARIMA-PSO-BPNN hybrid model are smaller than those of the SARIMA and SARIMA-BPNN hybrid models. In the test set, the MAE, MSE, RMSE, and MAPE values of the SARIMA-PSO-BPNN hybrid model were all smaller than those of the SARIMA and SARIMA-BPNN hybrid models (Table 3). For both the training and test sets, the predicted values from the SARIMA, SARIMA-BPNN, and SARIMA-PSO-BPNN hybrid models are strongly correlated with the observed values (Table 4). The performance of the three models in predicting the incidence of HFMD from January to December 2019 is shown in Fig. 7.

**Table 3:** Predictive performance of the three models in the training and test sets

Models	Training set				Test set			
	MAE	MSE	RMSE	MAPE	MAE	MSE	RMSE	MAPE
SARIMA	1.98	16.55	23.41	0.23	3.93	7.52	10.63	0.62
SARIMA-BPNN	1.11	12.91	18.25	0.13	0.96	2.41	3.41	0.08
SARIMA-PSO-BPNN	0.88	8.58	12.14	0.33	0.81	2.31	3.27	0.06

**Table 4:** The correlations between the predicted and observed values of the three models

Models	Training set		Test set	
	Pearson	Spearman	Pearson	Spearman
SARIMA	0.94	0.93	0.95	0.92
SARIMA-BPNN	0.98	0.99	0.99	1.00
SARIMA-PSO-BPNN	0.99	1.00	0.99	1.00

## Discussion

Our findings suggest that the incidence of HFMD in mainland China from January 2008 to December 2019 exhibited obvious seasonality, with a peak from May to June and a low peak from January to February, which is consistent with previous studies [2, 40, 41]. This is mainly because of two reasons. The first and most important reason for this is climatic factors. Numerous studies have indicated that the occurrence of HFMD is associated with meteorological factors, including temperature, humidity, sunshine, and wind speed [13, 42–44]. For example, the virulence and spread of enteroviruses under ambient conditions are mainly influenced by temperature and relative humidity, with a higher survival rate at 20 °C, and more likely to survive at 80% humidity [41]. A previous study found that an increase of 200 mm in rainfall was associated with a 19% increase in the risk of developing HFMD in Vietnam [45]. Wang et al. [42] found a positive correlation between wind speed, sunshine, and hospitalization due to HFMD. The main reason for this meteorological factor is that the vast territory of China spans a wide range of latitudes, most of which are located in the temperate zone, and some southern regions are located in subtropical and tropical regions, featuring a significant monsoon climate characterized by cold winters, hot summers, dry winters, and rainy summers [46].

Another significant factor influencing the development of HFMD is the holiday effect [47]. Previous studies have shown that holidays lasting more than a month may have a positive impact on reducing the transmission of HFMD from schools, whereas short holidays may have a limited impact on the transmission of HFMD [47, 48]. For primary and kindergarten schools in China, long public holidays occur twice a year, with annual winter and summer holidays. In general, winter holidays fall from January to February each year and summer holidays fall from July to August each year. These two public holidays last for approximately one-two months, offering relatively long holidays. Moreover, owing to seasonal changes in mainland China, meteorological factors such as temperature and humidity are beneficial for the transmission of HFMD between May and June each year [41]. Although primary and kindergarten schoolchildren have short holidays, such as May Day and the Dragon Boat Festival, which reduce the likelihood of their gathering, this short holiday has a limited impact on the transmission of HFMD in schools; instead, the incidence of HFMD was higher during this period.

Time-series data are collected at different times, which describe the changes in the state of something over time [49]. There are several time-series models, such as ARIMA, exponential smoothing, GARCH, VAR, and prophet models. However, ARIMA is one of the most classic time-series models and has been widely used to predict infectious diseases, including COVID-19[50], hepatitis B [28], tuberculosis [19], human brucellosis [51], HFMD [52], and pertussis [53]. SARIMA is a powerful forecasting tool in public health informatics [50] that provides an important reference for surveillance and early warning of infectious diseases. Several studies have confirmed that SARIMA achieves better predictive performance in forecasting the incidence of HFMD [16, 53–56]. This might be because of the capability of SARIMA models to effectively capture the nature of the dependency between current and past observations based on historical data while also considering the dynamic nature of infectious diseases [57]. Although the SARIMA model has many advantages in the prediction of infectious disease incidence, it still has some shortcomings. For example, it is incapable of dealing with the nonlinear part of the information in an infectious disease time series [24].

To overcome the shortcomings of the SARIMA model, machine-learning forecasting models can effectively extract nonlinear relationships from data [21]. Previous studies have confirmed that the BPNN approach achieves a superior performance in predicting the incidence of HFMD. Liu et al. [21] found that the multivariate BPNN model could effectively forecast the HFMD incidence series from 2009 to 2016 in Jiangsu Province, China, because of its robustness, fault tolerance, and adaptive learning ability. Li et al. [58] confirmed that the BPNN model had higher forecasting accuracy and more accurate predictions than the ARIMA model. Furthermore, the BPNN is not required to satisfy strict assumptions, and can satisfy both linear and nonlinear mappings to handle complicated and multivariate time-series issues [59]. Therefore, in this study, the BPNN model was used to construct a hybrid model for comparison with SARIMA as the baseline model.

However, the BPNN also has some disadvantages; for instance, it is prone to fall into local minimum values, slow convergence speed, and poor training efficiency of the network [60–63]. To overcome the shortcomings of the BPNN model, the PSO algorithm is introduced to construct the PSO-BPNN model. The PSO algorithm provides good global optimization capability by learning from population intelligence. The PSO algorithm was developed by optimizing the BPNN by replacing the gradient descent method to adjust the network weights and thresholds and to achieve an optimal BPNN model [60]. Therefore, the PSO-BPNN model combines the advantages of both the PSO algorithm and the BPNN model, and it can improve the accuracy of predictions, which has been confirmed in previous studies [60–62].

Therefore, we developed a basic SARIMA-BPNN model based on the SARIMA model. Then, we optimize the SARIMA-BPNN model with the PSO algorithm to obtain the optimal SARIMA-PSO-BPNN model. Compared to the results of similar studies, in the study, the MAPE value of the SARIMA-PSO-BPNN hybrid model in the test set(6%) was slightly lower than reported in the previous study(8.82%) [53]. Our results revealed that the prediction performance of the SARIMA-PSO-BPNN hybrid model (Table 4 and Fig. 7) outperformed the SARIMA and SARIMA-BPNN hybrid models, indicating that the SARIMA-PSO-BPNN hybrid model has a stronger generalization ability and provides excellent predictability. This may be because, in practice, the HFMD time series contains both linear and nonlinear complex time-series relationships [23]. The SARIMA model provides superior handling of linear information in the HFMD time series. The PSO algorithm optimizes the parameters of the SARIMA-BPNN hybrid model and improves the capability of the SARIMA-PSO-BPNN hybrid model to handle the complex time series relationships of HFMD; therefore, the SARIMA-PSO-BPNN hybrid model has a better generalization capability. These results are consistent with those of previous studies. Yu et al. [16] used SARIMA, NNAR, SARIMA-NNAR, and a wavelet-based SARIMA-NNAR hybrid model to predict the number of HFMD cases using data from 2009 to 2016 in Zhengzhou, China. Their study showed that the SARIMA-NNAR hybrid model demonstrated excellent prediction performance and was able to effectively forecast the incidence of HFMD. Zou et al. [23] indicated that the SARIMA-SVR hybrid model can accurately predict the incidence of HFMD and provide an effective decision-making tool for the prevention and control of HFMD in Wuhan, China.

However, our findings are inconsistent with those of previous studies. Yoshida et al. [64] used the LSTM model to forecast the incidence of HFMD in Japan, and their results showed that the LSTM approach could accurately estimate future epidemic patterns of HFMD in Japan. Meng et al. [65] used the XGBoost model and Random Forest model to predict the incidence of HFMD from January 2009 to December 2017 in mainland China and found that the XGBoost model was more suitable for predicting the incidence of HFMD in mainland China. Zhang et al. [52] used ARIMA and LSTM to forecast the incidence of HFMD in Ningbo, China, and their study indicated that LSTM forecasting performance was superior to that of the ARIMA model. These differing findings may be due to the different choices of study areas and time period of the research.

This study had several limitations. First, data for our study were obtained from the China Public Health Science Data Center. Although the data are authoritative, there may be under-reporting and misreporting of HFMD cases in mainland China. Second, overall data on the incidence of HFMD in 31 provinces and municipalities in mainland China were collected, and we were unable to independently collect data on HFMD in southern and northern mainland China. However, there was a difference between the northern and southern incidences of HFMD [66]; therefore, there was some bias in the prediction of HFMD incidence in our study. Third, HFMD occurs because of several factors, including meteorological [11, 13, 43] and holiday effect factors [47]. However, in this study, the meteorological and holiday effect factors were excluded from the prediction model. In future studies, we will attempt to individually collect HFMD incidence data from 31 provinces and municipalities in mainland China, while considering meteorological and holiday effect factors are taken into account in the prediction model to obtain more accurate prediction results for HFMD surveillance and early warning in mainland China.

## Conclusions

The present study found that the SARIMA-PSO-BPNN hybrid model overcomes the problem of insufficient optimization of the parameters of the traditional hybrid model, which improves the accuracy of prediction and provides an information reference for early warning and surveillance of HFMD in mainland China.

## Supplementary Information

**Additional file 1.**

## Data Availability

Data supporting the findings of this study are available from the China Public Health Science Data Center website (https://www.phsciencedata.cn/Share/index.jsp), the National Health Commission of the People’s Republic of China website (http://www.nhc.gov.cn/jkj/pgzdt/new_list.shtml), and the Chinese Statistical Yearbook website (http://www.stats.gov.cn/tjsj/ndsj/2021/indexch.htm) without restrictions.

## Abbreviations

HFMD: Hand, foot, and mouth disease
ARIMA: Auto-regressive integrated moving average
SARIMA: Seasonal auto-regressive integrated moving average
BPNN: Backward propagation neural network
AIC: Akaike information criterion
BIC: Bayesian Schwarz information criterion
AICc: Akaike Information Criterion, corrected
ADF: Augmented Dickey-Fuller
MAE: Mean absolute error
MSE: Mean square error
RMSE: Root mean square error
ACF: Auto-correlation function
PACF: Partial auto-correlation function
PSO: Particle swarm optimization
GARCH: Generalized autoregressive conditional heteroskedasticity
VAR: Vector auto-regression
NNAR: Neural network auto-regression
SVR: Support vector regression
XGBoost: Extreme gradient boosting
LSTM: Long short-term memory network
