# Supplementary material for: Research on hand, foot and mouth disease incidence forecasting using hybrid model in mainland China
Source: BMC Public Health. 2023 Mar 31;23:619. doi: 10.1186/s12889-023-15543-9 (PMC10064964; doi:10.1186/s12889-023-15543-9)
Supplement: Supplementary file 1 — Additional file 1. [file 12889_2023_15543_MOESM1_ESM.pdf]

Data on monthly HFMD incidence from January 2008 to December 2018 in 31 provinces and municipalities in mainland China

Date	HFMD incidence 1/100,000
2008/1	0.1211
2008/2	0.0285
2008/3	0.1082
2008/4	0.9867
2008/5	13.3209
2008/6	7.6841
2008/7	5.1179
2008/8	2.1166
2008/9	1.7976
2008/10	2.1454
2008/11	2.3918
2008/12	1.1872
2009/1	0.5657
2009/2	0.6195
2009/3	4.8130
2009/4	15.7863
2009/5	12.6347
2009/6	13.3622
2009/7	11.8276
2009/8	7.3072
2009/9	6.4129
2009/10	5.6876
2009/11	4.5499
2009/12	3.4445
2010/1	2.7987
2010/2	1.7275
2010/3	6.1908
2010/4	19.0673
2010/5	26.4549
2010/6	25.3625
2010/7	18.6953
2010/8	8.4167
2010/9	7.5358
2010/10	6.6139
2010/11	5.6971
2010/12	4.3994
2011/1	2.1195
2011/2	0.7956

2011/3	2.7465
2011/4	7.7386
2011/5	17.4660
2011/6	22.6272
2011/7	18.2078
2011/8	9.2708
2011/9	8.8315
2011/10	9.3601
2011/11	12.5882
2011/12	9.0399
2012/1	3.5274
2012/2	3.0886
2012/3	7.4647
2012/4	18.4700
2012/5	33.7441
2012/6	27.8138
2012/7	17.4746
2012/8	8.4553
2012/9	10.3073
2012/10	10.5850
2012/11	10.9402
2012/12	9.0924
2013/1	4.2064
2013/2	2.4564
2013/3	6.5525
2013/4	12.7396
2013/5	18.8719
2013/6	22.4087
2013/7	18.7874
2013/8	11.3891
2013/9	13.4647
2013/10	9.4211
2013/11	7.7591
2013/12	6.9743
2014/1	3.7636
2014/2	2.9993
2014/3	11.9901
2014/4	31.7321
2014/5	38.6478
2014/6	35.0081
2014/7	22.3476

2014/8	10.3420
2014/9	14.5413
2014/10	15.3154
2014/11	11.0490
2014/12	7.3202
2015/1	4.0942
2015/2	2.3204
2015/3	4.7426
2015/4	12.9312
2015/5	23.2992
2015/6	25.2986
2015/7	18.6310
2015/8	12.9544
2015/9	13.2855
2015/10	11.3507
2015/11	9.6428
2015/12	8.0490
2016/1	5.5243
2016/2	2.2401
2016/3	5.4329
2016/4	17.2251
2016/5	32.1052
2016/6	32.5296
2016/7	22.3260
2016/8	11.8574
2016/9	9.7790
2016/10	13.3622
2016/11	15.4771
2016/12	10.2974
2017/1	5.1744
2017/2	2.7598
2017/3	4.4556
2017/4	8.4711
2017/5	15.4558
2017/6	22.3412
2017/7	19.5816
2017/8	12.2141
2017/9	16.3145
2017/10	18.5765
2017/11	8.9940
2017/12	5.5002

2018/1	2.2782
2018/2	0.8984
2018/3	2.2424
2018/4	8.8346
2018/5	28.2647
2018/6	30.1643
2018/7	26.3648
2018/8	15.5764
2018/9	16.8543
2018/10	14.0791
2018/11	12.9040
2018/12	10.9518
2019/1	5.9341
2019/2	1.9996
2019/3	3.8252
2019/4	11.0416
2019/5	19.0507
2019/6	23.9272
2019/7	24.0781
2019/8	13.7035
2019/9	13.3664
2019/10	9.3081
2019/11	7.1032
2019/12	4.5292
